# Evaluating the role of IL-11, a novel cytokine in the IL-6 family, in a mouse model of spinal cord injury

**DOI:** 10.1186/1742-2094-9-134

**Published:** 2012-06-20

**Authors:** Newton Cho, Dung H Nguyen, Kajana Satkunendrarajah, Donald R Branch, Michael G Fehlings

**Affiliations:** 1 Department of Genetics and Development, Toronto Western Research Institute and Spinal Program, Krembil Neuroscience Center, University Health Network, 399 Bathurst Street, Toronto, ON, M5T 2S8, Canada; 2 Department of Surgery, University of Toronto, 100 College Street, Toronto, ON, M5G 1L5, Canada; 3 Research and Development, Canadian Blood Services, Departments of Medicine and Laboratory Medicine and Pathobiology, Division of Cell and Molecular Biology, Toronto General Research Institute, University of Toronto, 67 College Street, Toronto, ON, M5G 2M1, Canada; 4 Krembil Chair in Neural Repair and Regeneration, Toronto Western Hospital, University Health Network, 399 Bathurst Street, Toronto, ON, M5T 2S8, Canada

**Keywords:** Spinal cord injury, IL-11, mouse model, locomotor recovery, gp130 receptor, immune response, electrophysiology

## Abstract

**Background:**

Spinal cord injury (SCI) is a devastating condition with substantial functional and social morbidity. Previous research has established that the neuroinflammatory response plays a significant role in cord damage post-SCI. However, global immunosuppressive therapies have demonstrated mixed results. As a result, more specific therapies modulating inflammation after injury are needed. In this regard, research into cytokine signaling has demonstrated that cytokines of the gp130 family including IL-6 and leukemia inhibitory factor (LIF) play key roles in mediating damage to the spinal cord. Since members of the gp130 family all share a common signal transduction pathway via the JAK/STAT system, we performed the first study of a relatively new member of the gp130 family, IL-11, in SCI.

**Methods:**

A validated clip-compression mouse model of SCI was used to assess for temporal changes in expression of IL-11 and its receptor, IL-11Rα, post-SCI. To elucidate the role of IL-II in the pathophysiology of SCI, we compared differences in locomotor recovery (Basso Mouse Score; CatWalk), electrophysiological spinal cord signaling, histopathology, and the acute inflammatory neutrophil response in IL-11Rα knockouts with littermate wild-type C57BL/6 mice.

**Results:**

We found an increase in gene expression of IL-11 in the spinal cord to a peak at twenty-four hours post-SCI with increases in IL-11Rα gene expression, peaking at seven days post-SCI. In spite of clear changes in the temporal expression of both IL-11 and its receptor, we found that there were no significant differences in motor function, electrophysiological signaling, histopathology, or neutrophil infiltration into the spinal cord between wild-type and knockout mice.

**Conclusions:**

This is the first study to address IL-11 in SCI. This study provides evidence that IL-11 signaling may not play as significant a role in SCI as other gp130 cytokines, which will ideally guide future therapy design and the signaling pathways those therapies target.

## Background

Spinal cord injury (SCI) is a devastating condition, which is associated with severe physical burden in addition to psychological, social, and economic stress [1]. Despite improvements in the understanding of the pathophysiology of SCI, there are currently no robustly effective treatments to improve neurological function in this condition [1].

As a result, various methods to prevent and reduce morbidity and mortality after SCI have been or are in the process of being investigated. In this regard, research has demonstrated that SCI is a biphasic process consisting of a primary injury characterized by contusion, compression, or transection of the spinal cord followed by a secondary injury phase that exacerbates the initial primary insult including apoptosis, ischemia of the spinal cord, and inflammation [1-3]. Neuroinflammation, via cytokine signaling and cellular infiltration into the cord, plays a significant role in secondary damage following SCI [3]. Hence, therapies aimed at mitigating the neuroinflammatory response have logically been candidates for spinal cord neuroprotection and repair. Global immunosuppressive therapies such as methylprednisolone have been attempted but with mixed results [3-5].

More specific therapies aimed at inflammation post-SCI are thus needed. In this respect, to further understand the signaling dynamics of inflammation in mediating damage after SCI, the gp130 receptor family of cytokines has been the subject of an increasing amount of research. This family of cytokines all shares the gp130 membrane glycoprotein as a common signal transducer, which then results in signal transduction via the Janus kinase/signal transducers and activators of transcription (JAK/STAT) pathway [6]. This family of cytokines includes IL-6 and leukemia inhibitory factor (LIF), which have been shown to play pivotal roles in SCI. IL-6 has been shown to play a central role in mediating pro-inflammatory damage after SCI [7-9]. LIF has been shown to have both pro-inflammatory and oligodendrocyte preservation effects after SCI [10-12]. STAT3 has also been shown to play an important role in regulating reactive astrocyte migration and responses after SCI [13,14]. This body of evidence suggests that the gp130 receptor and its associated cytokines and signaling pathways play an important role in SCI.

To further understand cytokine signaling in this family and SCI, a relatively novel member of the gp130 family, IL-11, was addressed in this study. IL-11 interacts with its ligand-specific cell surface receptor subunit IL-11Rα that then couples to gp130 and results in activation of JAK/STAT signaling [15]. *In situ* hybridization has demonstrated IL-11 expression in the hippocampus and ventral area of the spinal cord [15]. IL-11 has also been shown to play an immunomodulatory role in an experimental autoimmune encephalomyelitis (EAE) model for multiple sclerosis with increased inflammation, demyelination, and oligodendrocyte and neuronal loss in IL-11Rα-deficient mice [16]. Moreover, IL-11 has been shown to play a role in mediating oligodendrocyte viability and maturation in primary human fetal oligodendrocyte-enriched cultures [17]. No previous studies to date have addressed the role of IL-11 in SCI.

With the limited number of studies of IL-11 in the central nervous system, we hypothesized that IL-11 would play an anti-inflammatory role and a role in oligodendrocyte preservation after SCI, resulting in worsened neurobehavioral outcomes with decreases in IL-11 signaling. We thus subjected IL-11Rα-deficient mice and wild-type littermate mice to SCI using a validated clip compression injury model [18-20] and assessed for differences in motor function, histopathology, electrophysiological signaling, and neutrophil infiltration in the spinal cord. This study is the first study to address the role of IL-11 in SCI and hopefully will provide a basis for future research in understanding the role of the gp130 family of cytokines in inflammation after injury. Ultimately, this understanding will hopefully lead to better therapies aimed at modulating cytokine signaling and decreasing damaging inflammatory processes after SCI.

## Methods

### Animals used and experimental groups

All experimental protocols were approved by the Animal Care Committee at the Toronto Western Research Institute in accordance with the policies of the *Guide to the Care and Use of Experimental Animals* as per the Canadian Council of Animal Care.

Female, adult wild-type C57BL/6 mice (15 to 20 g; Jackson Laboratories, Bar Harbor, ME, USA) aged eight to twelve weeks and littermate IL-11Rα knockout (IL-11Rα-deficient) mice fully congenic on a C57BL/6 background (strain B6.129S1-Il11ra1tm1Wehi/J; Jackson Laboratories) were used for behavioral analysis, histology, electrophysiology, and assessment of the inflammatory response after SCI. All genotyping was performed by Jackson Laboratories before delivering mice to our site. Wild-type female, adult C57BL/6 mice of similar weight and age were also purchased separately (Jackson Laboratories) for RT-PCR and ELISA analyses.

The overall study divided mice into two broad cohorts: 1) analysis of the gene and protein changes after SCI of IL-11 and IL-11Rα and 2) comparison of wild-type and IL-11Rα-deficient mice in terms of functional, histological, and inflammatory outcomes after SCI. To meet the first objective, a total of 65 wild-type C57BL/6 mice were used. To study the temporal changes in gene expression of IL-11 and IL-11Rα after SCI, animals were divided into six groups: uninjured control (n = 5), four hours post-SCI (n = 5), eight hours post-SCI (n = 5), twenty-four hours post-SCI (n = 5), three days post-SCI (n = 5), and seven days post-SCI (n = 5). To study the temporal changes in protein expression of IL-11 after SCI, animals were again divided into six groups: uninjured control (n = 5), four hours post-SCI (n = 6), eight hours post-SCI (n = 6), twenty-four hours post-SCI (n = 6), three days post-SCI (n = 6), and seven days post-SCI (n = 6). To meet the second objective, two groups of animals were used (total n = 47): IL-11Rα-deficient C57BL/6 mice (n = 11) and wild-type littermate C57BL/6 mice (n = 13) for behavioral analysis. The behavior of these mice was followed for six weeks followed by electrophysiological recordings of these mice at six weeks after which the mice were sacrificed and then analyzed for histological characterization. Another set of uninjured IL-11Rα-deficient mice (n = 4) and uninjured wild-type littermate mice (n = 4) were used for electrophysiological recordings as control animals. Finally, another set of injured IL-11Rα-deficient mice (n = 5) and injured wild-type littermate mice (n = 5) and a separate set of uninjured control wild-type C57BL/6 mice (n = 5) were used to compare the inflammatory response between these mice after SCI.

### Spinal cord injury

Anesthesia in the mice was first induced with a mixture of isoflurane (1 to 4%), nitrous oxide, and oxygen (1:1/minute) in a chamber and then maintained using a mask integrated in a stereotaxic surgical frame. Surgery did not commence until the mice were no longer responsive to a nociceptive stimulus. Before surgery, a subcutaneous bolus of 0.5 mL of saline was given, and for the duration of the surgery, mice were placed on a heating pad at 37°C. Under aseptic conditions, the hair on the skin on the back of the mouse was shaved off and disinfected with a 1:1 mixture of 70% alcohol and iodine. A midline skin incision was then made to expose the superficial back muscle layers. The muscle attached to the vertebrae was dissected away using a No. 15 scalpel blade to expose the T7-T9 vertebrae. Laminectomy forceps were used to perform a laminectomy at T7-T9, and a dissecting hook was used to gently clear an extradural path on the ventral side of the spinal cord between T7-T8 without damaging the underlying spinal cord. The spinal cord was compressed extradurally at T7-T8 ventrally and dorsally using a modified aneurysm clip (Fejota^TM^ mouse clip; University Health Network, Toronto, Canada) that was passed around the spinal cord in the path created using the dissecting hook. The clip was released rapidly with a closing force of 8.5 grams and was allowed to compress the cord for 30 seconds before being removed, resulting in a moderate SCI. Surgeries involving knockout mice and their wild-type littermates were performed in alternation. Mice to be used in long-term behavioral characterization also had a piece of sterile absorbable gelatin sponge (Gelfoam; Pfizer Inc., New York, NY, USA) placed over the dura between T7-T9 to avoid excessive scar formation over the cord in the long term. Uninjured control mice did not receive any injury or laminectomy.

After completion of the surgery, the superficial muscle layers were sutured together followed by closing of the skin incision using small Michel clips. Antibiotic cream (Polysporin; Johnson & Johnson Inc., New Brunswick, NJ, USA) was then generously applied over the area of the skin incision. 1.0 mL of saline was also immediately administered subcutaneously to replace blood volume lost during the procedure as well as 0.1 mL of buprenorphine (0.05 mg/kg) subcutaneously to alleviate post-operative pain. During recovery from anesthesia, mice were also placed in new sterile cages under a heating lamp and eventually housed in a temperature-controlled room at 27°C for as long as required during experimentation. Food and water were provided ad libitum, and the mice received antibiotic in their drinking water (Clavamox drops; Pfizer Animal Health, New York, NY, USA). Buprenorphine was subcutaneously administered twice daily for three more days after surgery. Bladders were manually voided twice daily until bladder function returned.

### Real-time PCR (RT-PCR)

Mice were overdosed with isoflurane. Each animal was then perfused intracardially with 30 mL of RNase-free Ringer’s solution at 4°C. The spinal column was then dissected out of the mouse and immediately placed in RNase-free Ringer’s solution at 4°C. A spinal cord section of 0.5 cm centering on the lesion epicenter was then isolated after removal of dura mater around the cord, and the cord was frozen on dry ice. The spinal cord samples were then manually ground and homogenized in Trizol (Invitrogen Co., Carlsbad, CA, USA), and RNA extraction was completed according to the manufacturer’s instructions (Qiagen Inc., Toronto, Canada). RNA concentration was determined using the NanoDrop spectrophotometer (Thermo Fisher Scientific, Wilmington, DE, USA). cDNA synthesis was then performed using the SuperScript III Reverse Transcriptase kit according to the manufacturer’s instructions (Invitrogen Co.). RT-PCR was then carried out on a 396-well plate with each well containing cDNA, appropriate forward and reverse primers (Table 1) for IL-11, IL-11Rα, and HPRT (Integrated DNA Technologies Inc., Coralville, IA, USA), nuclease-free water, and SYBR Green (Applied Biosystems, Foster City, CA, USA). Each sample was run in triplicates for each gene, and fold change analysis was standardized to *hypoxanthine phosphoribosyltransferase* (HPRT).

**Table 1:** Sequences of primers used to amplify mouse IL-11, IL-11Rα, and HPRT

Primer name	Sequence
Forward IL-11	5′-AAT TCC CAG CTG ACG GAG ATC ACA-3′
Reverse IL-11	5′-TCT ACT CGA AGC CTT GTC AGC ACA-3′
Forward IL-11Rα	5′-TGG AAG TCC ACC TGA GGA ATG TGT-3′
Reverse IL-11Rα	5′-AGA CCG CAC ACA CTC TCC AAT CAT-3′
Forward HPRT	5′-AGG AGT CCT GTT GAT GTT GCC AGT-3′
Reverse HPRT	5′-GGG ACG CAG CAA CTG ACA TTT CTA-3′

### ELISA

Mice were overdosed with isoflurane. Each animal was then perfused intracardially with 30 mL of saline (0.9% NaCl) at 4°C. The spinal column was then dissected out of the mouse and immediately placed in saline at 4°C. A spinal cord section of 0.5 cm centering on the lesion epicenter was then isolated after removal of dura mater around the cord, and the cord was frozen on dry ice. The spinal cord samples were then manually ground and homogenized in RIPA buffer containing a cocktail of EDTA and protease inhibitors (5 mM Tris-HCl, 4 mM EDTA, 1 μM pepstatin, 100 μM leupeptin, 100 μM phenylmethylsulfonyl fluoride, and 10 μg/mL aprotinin) at 4°C. Total protein concentrations of each of the samples was then determined via the Lowry method. A 96-well ELISA plate (Nunc-Immuno Plates MaxiSorp; Thermo Fisher Scientific, Rochester, NY, USA) was then prepared and samples and reagents added as per manufacturer’s instructions for the mouse IL-11 DuoSet ELISA Development System kit (catalog number DY418; R & D Systems Inc., Minneapolis, MN, USA). Each sample was run in duplicate.

### Assessment of motor function recovery

Motor function recovery comparisons between wild-type and IL-11Rα-deficient mice were performed using two motor tests: Basso Mouse Scale (BMS) and the CatWalk gait analysis system.

The Basso Mouse Scale (BMS) is a validated scale to monitor the progress of hindlimb functional recovery after SCI [21]. The scale extends from 0 to 9 (0 = no ankle movement and 9 = normal locomotion) and involved the observation of each mouse in an open field for four minutes by two observers. The observers were blinded to the genetic identity of each mouse. Each hindlimb was scored individually, and the scores of each hindlimb were then averaged to create a score for the entire animal. Animals were observed at three days post-injury and then seven days post-injury and, from then on, observed weekly for a total of six weeks post-injury. Subscore analysis was also performed at the above time points for animals that achieved frequent plantar stepping (BMS score of 5 and above). The BMS subscore scale extends from 0 to 11 and is generally used to detect differences between animals that have reached a plateau at similar levels on the main BMS [21]. Animals that had not reached frequent plantar stepping were scored as 0 according to the subscore scale.

Gait analysis using the CatWalk gait analysis system was performed at six weeks post-injury to further compare wild-type with IL-11Rα-deficient mice. The CatWalk gait analysis system assesses gait parameters such as stride length, regularity index (measure of coordination), print area, swing speed, and base of support that are not assessed by the Basso Mouse Scale, providing a further level of characterization of any gait differences and functional recovery between wild-type and IL-11Rα-deficient mice [22]. Only animals that had reached frequent plantar stepping on the Basso Mouse Scale were analyzed using the CatWalk gait analysis system as the system requires the mouse to step across a platform for the analysis.

### Sensory evoked potential recordings

Six weeks post-injury, sensory evoked potentials (SEPs) were recorded from 18 mice (uninjured IL-11Rα-deficient mice, n = 4; uninjured wild-type C57BL/6 mice, n = 4; injured IL-11Rα-deficient mice, n = 5; injured wild-type C57BL/6 mice, n = 5). The evoked potentials were recorded using stainless steel needle electrodes and a Keypoint Portable System. Mice were anesthetized using a mixture of isoflurane (1 to 4%), nitrous oxide and oxygen (1:1/minute) in a chamber and then maintained using a mask integrated in a stereotaxic frame. The recording electrodes were positioned extradurally over the spinal cord at C2 and C3. Two stainless steel recording electrodes were inserted into the hindpaw. A ground electrode was inserted subcutaneously between the stimulating and recording electrodes. A constant current stimulus of 0.1 ms duration and 2.0 mA intensity was applied at a rate of 5.7 Hz to the hindpaw. At a bandwidth of 10 to 3000 Hz, a total of 300 traces were averaged and replicated. SEP peak latency was measured from the start of the stimulus (S) to the peak of the first positive peak (P1).

### Histopathology

Mice were overdosed with isoflurane. The mice were then perfused intracardially with 10 mL of phosphate-buffered saline (PBS) followed by 30 mL of paraformaldehyde (4% w/v in PBS, pH 7.4) at 4°C. The spinal cords were then dissected out from each mouse and post-fixed overnight in 10% sucrose/4% paraformaldehyde solution at 4°C. The spinal cords were then cryoprotected by placing the cords in 20% sucrose solution in PBS for 24 hours at 4°C. A 1.0 cm length of spinal cord centered at the injury epicenter was then embedded in Tissue-Tek Optimal Cutting Temperature compound (OCT; Sakura Finetek USA Inc., Torrance, CA, USA) and stored at −80°C.

Serial transverse sections of 20 μm thickness were then prepared for each spinal cord to be analyzed and mounted on glass slides and stored at −80°C. A total of five injured knockout mice and five injured littermate wild-type mice at six weeks post-injury were analyzed and compared. Serial spinal cord sections spaced 120 μm apart were stained with Luxol Fast Blue (LFB) and hematoxylin/eosin (H and E) for each animal spanning 600 μm rostral and caudal to the injury epicenter. Tissue sections were first dried at room temperature for one hour and submerged in PBS for one minute to remove any excess OCT. The sections were then dipped in 50% ethanol for one minute and then placed in LFB overnight at 56°C. The following day, the sections were then dipped in 95% ethanol for five minutes followed by ddH_2_O for five minutes. The sections were then destained in 0.05% LiCO_2_ followed by a dip in 70% ethanol for 30 seconds. The sections were then again placed in ddH_2_O for five minutes. After, the sections were dipped in hematoxylin for 20 minutes followed by a rinsing step with tap water for 10 minutes. The sections were then dipped in eosin for five seconds followed by two five-minute dips in 95% ethanol and two five-minute dips in 100% ethanol. Finally, the sections were subjected to three five-minute dips in xylene and then cover slipped.

The injury epicenter was defined as the section with the largest area of scar/vacuolation tissue and least amount of grey matter. Tissue sections were analyzed for scar (defined as fibrous and inconsistent tissue matrix), grey matter sparing (stained red due to eosin), and white matter sparing (stained blue due to LFB). Tissue sections were analyzed using the Cavalieri Probe (Stereo Investigator 64-bit software; MBF Bioscience, Williston, VT, USA) and areas of scarring, grey matter, and white matter were expressed as percentages of the total area of each tissue section. Experimenters were blinded to the genetic identity of the tissue sections.

### Myeloperoxidase activity assay

Myeloperoxidase (MPO) activity was assayed using a myeloperoxidase fluorometric detection kit according to the manufacturer’s instructions (catalog number ADI-907-029; Enzo Life Sciences International Inc., Plymouth Meeting, PA, USA). Five wild-type and five knockout mice were injured and their spinal cords extracted at 24 hours post-injury. Five uninjured wild-type mice spinal cords were also assayed to serve as a control for any aberrant MPO activity that may be detected in the cords due to methodological error. MPO is present in the azurophilic granules of polymorphonuclear leukocytes and is unique to neutrophils and monocytes. The time point of 24 hours post-SCI was chosen given that neutrophil infiltration into the spinal cord peaks at this time after SCI in the mouse and rat [23]. Briefly, the mice were overdosed with isoflurane. The mice were then perfused intracardially with 30 mL of saline (0.9% NaCl) at 4°C. The spinal column was then dissected out of each mouse and immediately placed in saline at 4°C. A spinal cord section of 0.5 cm centering on the lesion epicenter was then isolated after removal of dura mater around the cord, and the cord was frozen on dry ice. The spinal cord samples were then manually ground and homogenized in assay buffer provided in the MPO detection kit containing 10 mM of N-ethylmaleimide (NEM). The samples were centrifuged at 12 000 g for 20 minutes at 4°C and the supernatant removed and discarded. The pellet was then resuspended in assay buffer with 10 mM NEM and 0.5% w/v hexadecyltrimethylammonium (HTA-Br), homogenized, and subjected to two freeze-thaw cycles. The samples were then centrifuged at 8 000 g for 20 minutes at 4°C. The remaining supernatant of each sample was then loaded into a 96-well plate with detection reagent as specified by the manufacturer and run in duplicate. The fluorometric reaction was allowed to proceed for 60 minutes, and the fluorescence was measured at an excitation wavelength of 530 to 570 nm and emission wavelength at 590 to 600 nm.

### Statistical analysis

All data are presented as mean ± standard error of the mean (SEM). Gene and protein expression data, electrophysiology latency data, and myeloperoxidase activity data was analyzed using a one-way analysis of variance (ANOVA). The Basso Mouse Scale and histology data was analyzed using a two-way analysis of variance (ANOVA) repeated measures. Significant ANOVA results were followed by post hoc Bonferroni analysis. Pair-wise comparisons between wild-type and knockout mice for CatWalk data were performed using Student’s t test. The significance level of all analyses was set at *P* <0.05.

## Results

### IL-11 and IL-11Rα expression change after spinal cord injury

**Figure 1:**
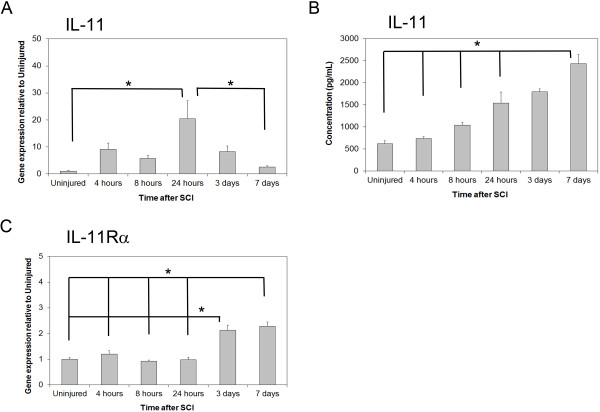
**IL-11 and IL-11Rα expression is increased after spinal cord injury in wild-type mice.** qRT-PCR for temporal gene expression of (**A**) IL-11 in the spinal cord after SCI demonstrate a peak in gene expression at 24 hours, supported by increasing (**B**) IL-11 protein levels in the spinal cord as measured via ELISA. qRT-PCR for temporal gene expression of (**C**) IL-11Rα also revealed an increase in gene expression with time after injury with peak expression occurring later than IL-11. Data represent mean ± SEM (error bars), n = 5 for each uninjured group, n = 5 for time points of qRT-PCR, n = 6 for time points of ELISA. *P <0.05 (one-way ANOVA followed by post hoc Bonferroni test).

To establish whether IL-11 and its associated receptor IL-11Rα respond to SCI, RT-PCR and ELISA were used to assess for changes in gene and protein expression at various times post-injury. Any potential role for IL-11 in SCI would logically be reflected in accompanying changes in gene and protein expression post-injury. RT-PCR analysis revealed that IL-11 gene expression changes significantly after SCI (one-way ANOVA, *P* = 0.002; Figure 1A). Post hoc analysis revealed that IL-11 gene expression at 24 hours was significantly different from uninjured control mice (*P* = 0.002) and gene expression at seven days (*P* = 0.005) while no other time points were significantly different from uninjured mice in the post hoc analysis, suggesting peak gene expression at 24 hours. IL-11 gene expression changes were corroborated with ELISA data demonstrating a steady increase in the amount of IL-11 present in the spinal cord with time post-SCI (one-way ANOVA, *P* <0.001; Figure 1B). Post hoc analysis revealed peak levels of IL-11 at seven days post-SCI with significantly greater amount of IL-11 compared to uninjured control mice (P <0.001), mice at four hours (*P* <0.001), eight hours (*P* <0.001), and twenty-four hours (*P* = 0.002) post-SCI. Significant IL-11Rα gene expression changes also occurred post-injury (one-way ANOVA, *P* <0.001; Figure 1C). The pattern of increase in gene expression of IL-11Rα differed from that of IL-11 with post hoc analysis revealing that gene expression was significantly greater at three days and seven days relative to the other time points and the uninjured control mice (*P* ≤0.001 relative to other time points for both three days and seven days post-SCI). Moreover, the magnitude of the fold change in gene expression differed between IL-11 and IL-11Rα post-injury. IL-11 demonstrated a peak gene expression fold change at twenty-four hours post-injury relative to uninjured control mice of 20.41 ± 6.75 while IL-11Rα demonstrated a fold change at three and seven days post-injury relative to uninjured control mice of only 2.12 ± 0.20 and 2.29 ± 0.16, respectively.

### No difference between wild-type and IL-11Rα-deficient mice was observed in hindlimb motor recovery

**Figure 2:**
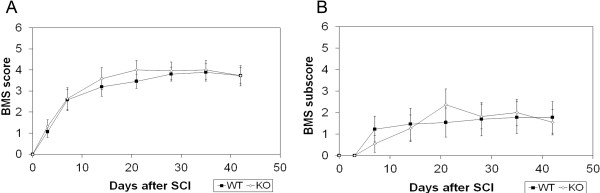
**Hindlimb functional recovery is not significantly different between wild-type and IL-11Rα-deficient mice.** (**A**) BMS and (**B**) BMS subscore analysis revealed no significant differences between wild-type (n = 13) and IL-11Rα-deficient (n = 11) mice in hindlimb motor recovery at the indicated time points up to six weeks (forty-two days) post-SCI (two-way repeated measures ANOVA). Data represent mean ± SEM (error bars).

Wild-type and knockout mice were observed for locomotor recovery for six weeks post-injury using the Basso Mouse Scale [21]. No significant differences in hindlimb function as assessed using the BMS were found over six weeks (two-way ANOVA repeated measures, *P* = 0.502; Figure 2A). Functional improvement in both the wild-type and knockout mice occurred until about twenty-one days (three weeks) post-injury when recovery plateaued. BMS subscore analysis also did not reveal any differences between the wild-type and knockout mice over six weeks (two-way ANOVA repeated measures, *P* = 0.582; Figure 2B). The BMS subscores plateaued relatively quickly at approximately fourteen days (two weeks) post-injury. This was likely due to the fact that the number of mice in both the wild-type and knockout group who reached frequent plantar stepping and thus were scored using the BMS subscore scale remained constant from that time after injury for the duration of the study, thus resulting in very little changes in the overall BMS subscore scale scores.

**Figure 3:**
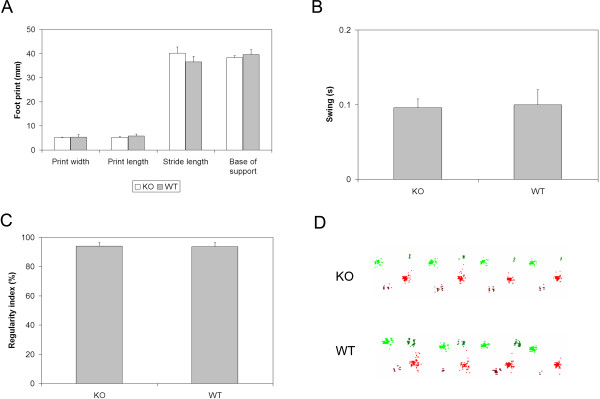
**CatWalk gait analysis at six weeks (forty-two days) post-SCI revealed no significant difference between injured wild-type (n = 5) and injured IL-11Rα-deficient (n = 5) mice in hindlimb.** (**A**) Print width, print length, stride length, base of support, (**B**) swing time, and (**C**) regularity index (Student’s t test). (**D**) Representative footprints from injured wild-type and injured knockout mice six weeks (forty-two days) post-SCI. Data represent mean ± SEM (error bars).

CatWalk gait analysis was also performed at six weeks post-SCI on mice that had received frequent plantar stepping on the BMS (score of 5 and above). The same number of wild-type and knockout mice (n = 5) achieved frequent plantar stepping at six weeks post-SCI, and there were no significant differences in the BMS scores between these two groups of mice (data not shown). This allowed for any true differences in gait using the CatWalk analysis to be determined without any systematic bias due to differences in gross hindlimb function as measured using the BMS. The CatWalk gait analysis system provided another degree of characterization of hindlimb motor recovery by assessing parameters not addressed in the BMS [22]. Comparison of the gait between wild-type and knockout mice revealed no differences in hindlimb print width (Student’s t test, *P* = 0.834), print length (Student’s t test, *P* = 0.522), stride length (Student’s t test, *P* = 0.409), and base of support (Student’s t test, *P* = 0.633; Figure 3A). There were also no significant differences in swing time (Student’s t test, *P* = 0.889; Figure 3B) and regularity index (Student’s t test, *P* = 0.900; Figure 3C), which is a measure for interlimb coordination. Figure 3D provides representative images of the footprints of the wild-type and knockout mice.

### Axonal conduction was not significantly different between wild-type and IL-11Rα-deficient mice following SCI

**Figure 4:**
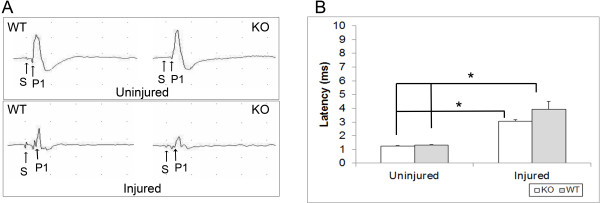
**Sensory evoked potential (SEP) latencies demonstrated no significant difference between injured wild-type (n = 5) and injured IL-11Rα-deficient mice (n = 5).** (**A**) Representative average waveforms are provided for injured and uninjured wild-type and IL-11Rα-deficient mice showing point of stimulation (S) and first positive peak (P1). (**B**) Both wild-type and knockout injured mice demonstrate significantly greater latencies compared to both uninjured wild-type (n = 4) and uninjured knockout (n = 4) control mice while uninjured wild-type and uninjured knockout mice do not demonstrate significant differences in latency from each other. Data represent mean ± SEM (error bars). *P <0.05 (one-way ANOVA followed by post hoc Bonferroni test).

Sensory evoked potentials were recorded to assess axonal function following injury in both wild-type (n = 5) and IL-11Rα-deficient mice (n = 5) using the mice analyzed in the CatWalk gait analysis component of the study. In addition, evoked potentials were also recorded from uninjured wild-type (n = 4) and IL-11Rα-deficient mice (n = 4) to control for any inherent differences in axonal conduction between wild-type and knockout mice. One-way ANOVA analysis revealed that there were significant differences in P1 peak latencies between the four groups overall (*P* <0.0001, Figure 4). However, post hoc analysis revealed no difference in peak latencies between uninjured wild-type and IL-11Rα-deficient mice (*P* = 1.000, Figure 4), suggesting no inherent differences in axonal conduction between wild-type and knockout mice. P1 latency was 1.31 ± 0.043 ms and 1.25 ± 0.029 ms in uninjured wild-type and uninjured IL-11Rα-deficient mice, respectively. However, both injured wild-type (*P* <0.0001 relative to uninjured wild-type and uninjured knockout) and injured IL-11Rα-deficient mice (*P* <0.05 relative to uninjured wild-type and uninjured knockout) showed significant increases in P1 latency indicating impaired axonal conduction. In wild-type mice, the P1 latency was increased to 3.94 ± 0.577 ms at six weeks after SCI while the P1 latency was increased to 3.04 ± 0.112 ms in IL-11Rα-deficient mice. Although SCI resulted in impaired axonal conduction in both the wild-type and IL-11Rα-deficient mice, there was no significant difference in axonal conduction following injury between wild-type and IL-11Rα-deficient mice (*P* = 0.376, Figure 4). The prominent peak recorded was abolished following spinal cord transection below the recording electrodes, indicating the spinal cord origin of the recorded signals.

### Spared tissue and lesion size did not differ between wild-type and IL-11Rα-deficient mice

**Figure 5:**
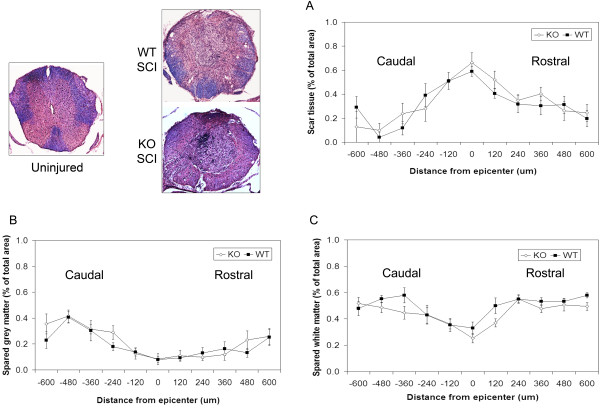
**No significant differences in histopathology were observed at six weeks post-SCI between injured wild-type (n = 5) and injured IL-11Rα-deficient (n = 5) mice.** Histopathological analysis of spinal cord sections from both wild-type and IL-11Rα-deficient mice revealed no significant differences in (**A**) scar tissue area, (**B**) grey matter area preservation, and (**C**) white matter area preservation (two-way repeated measures ANOVA). Data represent mean ± SEM (error bars).

Spinal cord sections spaced 120 μm apart spanning 600 μm on each side of the injury epicenter were analyzed for the five wild-type and five knockout mice used in the CatWalk gait analysis component of the study to assess for any possible histopathological differences due to changes in IL-11 signaling six weeks post-SCI, despite the lack of differences in hindlimb motor recovery and evoked potentials. Unlike rat models of SCI, no prominent cavitation was observed in the injured mouse spinal cords analyzed in this study. Instead, the injured mouse spinal cord lesions exhibited prominent disruption of the tissue matrix and scar formation which included most of the grey matter while sparing a rim of white matter. Hematoxylin and eosin staining with LFB of spinal cord sections with Cavalieri probing using Stereo Investigator software was used to calculate areas of scar formation, grey matter preservation, and white matter preservation in successive spinal cord sections of each animal analyzed. No significant differences between wild-type and knockout mice were found for area of scar (two-way ANOVA repeated measures, *P* = 0.738), grey matter area preservation (two-way ANOVA repeated measures, *P* = 0.540), and white matter area preservation (two-way ANOVA repeated measures, *P* = 0.104) as seen in Figure 5.

### Myeloperoxidase activity in the injured spinal cord is not different between wild-type and knockout mice

**Figure 6:**
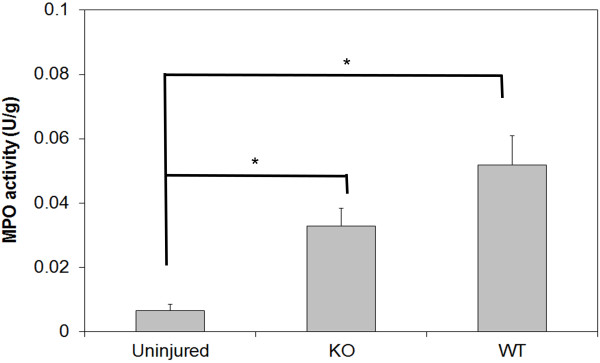
**Early neutrophil infiltration into the injured spinal cord does not appear to differ between wild-type and IL-11Rα-deficient mice.** Myeloperoxidase (MPO) activity in the spinal cord is not significantly different between injured wild-type (n = 5) and injured IL-11Rα-deficient (n = 5) mice 24 hours post-SCI, although injured mice demonstrate significantly greater MPO activity compared to uninjured wild-type control mice (n = 5). Data represent mean ± SEM (error bars). *P <0.05 (one-way ANOVA followed by post hoc Bonferroni test).

To assess for possible effects on inflammation that may have not manifested as significant neurobehavioral, electrophysiological, or histopathological outcomes, myeloperoxidase (MPO) activity in the spinal cord of wild-type and knockout mice was compared 24 hours post-SCI (Figure 6). Five wild-type and five knockout mice were compared for MPO activity. Five uninjured control wild-type mice were also analyzed to serve as a control for any aberrant methodological errors that may have affected the assay and resulted in inaccurate MPO readings. Both the knockout (0.032 ± 0.005 U/g, *P* <0.05) and wild-type (0.052 ± 0.009 U/g, *P* <0.001) injured mice had significantly elevated MPO levels in their spinal cords relative to the uninjured mice (0.007 ± 0.002 U/g), as expected (one-way ANOVA *P* <0.001). However, no significant difference was observed between the knockout and wild-type injured mice from each other after post hoc analysis (*P* = 0.157).

## Discussion

Previous research has demonstrated that neuroinflammation plays a prominent role in the secondary damage that occurs to the spinal cord after injury. In this regard, members of the gp130 family of cytokines (including IL-6 and LIF) and its associated JAK/STAT signaling pathway have been studied previously and have been shown to play important roles in inflammation and functional recovery after SCI [7-14]. IL-11, a newer member of the gp130 family of cytokines, has recently been shown to play an anti-inflammatory and neuroprotective role in multiple sclerosis [16] and a role in mediating oligodendrocyte viability and maturation in enriched primary oligodendrocyte human fetal cultures [17]. Previous research in other systems has also pointed to an anti-inflammatory role of IL-11. IL-11 has been shown to play an anti-inflammatory role in the airways and asthma [24]. Recombinant human IL-11 administration to activated macrophages *in vitro* has been shown to inhibit TNFα, IL-1β, IL-12, and nitric oxide production [15]. IL-11 has also been shown to play a role in decreasing mucosal damage in inflammatory bowel disease [25] and decreasing the severity of acute necrotizing pancreatitis [26]. Given the significant role of other cytokines in the gp130 family and evidence pointing to an anti-inflammatory role and effect on promoting oligodendrocyte survival, IL-11 was addressed in this study. We hypothesized that knocking out IL-11Rα, and thus eliminating IL-11 signaling, would result in decreased functional recovery and poorer histopathological outcomes due to increased inflammation after injury and poor oligodendrocyte survival. In contrast to our initial hypothesis, we found that knocking out IL-11Rα did not result in significant differences in functional recovery and histopathology after SCI.

While our results indicate that IL-11 is not a dominant player in the pathophysiology of SCI, our findings do not necessarily indicate that IL-11 signaling does not play any role in this condition. We did demonstrate for the first time that SCI affects the expression of IL-11 and its receptor, IL-11Rα, in spinal cord tissue. Previous research has demonstrated that IL-11 must first bind to IL-11Rα, and this complex of cytokine and receptor subunit then recruits the signaling gp130 receptor subunit [27]. This is important given that, while gp130 is ubiquitously expressed, the expression of α-receptors is restricted and tightly regulated, which limits the types of cells that respond to different members of the gp130 family [27].

In the current paper, we demonstrate that both IL-11 and IL-11Rα expression in the spinal cord change in response to injury thus suggesting that IL-11 and IL-11Rα play a role in SCI at the level of the spinal cord tissue. Interestingly, the gene expression profile of IL-11 differs from its receptor both in magnitude and in its temporal pattern with peak gene expression of IL-11 at approximately 24 hours post-SCI (twentyfold increase) while IL-11Rα gene expression peaks much later (twofold increase). To date, we have not come across many studies of gp130 cytokines that have documented the expression patterns of both the cytokine and its associated α-receptor in SCI, making it difficult to compare with other members in the family. However, given the necessity of the α-receptor in signaling, it is reasonable to propose that the expression of the α-receptor acts as the limiting factor in IL-11 signaling. As a result, given that the peak in IL-11Rα expression is on the order of days after SCI, this suggests that IL-11 signaling may not play as significant a role in the acute inflammatory response after injury, but more in the long-term sequelae such as oligodendrocyte survival. The possibility that IL-11 signaling may not play as significant a role in the acute inflammatory response was demonstrated in the data, with no significant differences between wild-type and knockout injured mice in myeloperoxidase activity levels at 24 hours post-SCI, when neutrophil infiltration into the cord is at its peak [23]. TNFα and IL-1β both have been shown to recruit neutrophils and macrophages into the spinal cord [23]. TNFα and IL-1β gene expression in the spinal cord have been shown to peak at approximately one hour and twelve hours, respectively, post-SCI with decreases in expression to basal levels by seven days in the mouse except for TNFα, which appears to peak again at fourteen and twenty-eight days post-SCI [28]. Therefore, despite evidence that IL-11 negatively regulates these cytokines [29], IL-11 signaling in the spinal cord *in vivo* after injury falls outside the appropriate window to have a significant effect on their expression in the early acute phases after injury to affect the recruitment of inflammatory cells such as neutrophils.

As a result, despite clear changes in gene and protein expression of IL-11 and IL-11Rα, we found that there were no significant differences in functional recovery between injured wild-type and injured IL-11Rα knockout mice. There were no significant differences in both the BMS hindlimb score and BMS subscore analysis between wild-type and knockout mice up to six weeks post-SCI. Further, hindlimb motor function was assessed at six weeks using the CatWalk gait analysis system. The CatWalk gait analysis system assesses motor parameters not detected using the BMS, and again, no significant differences were detected in print width, print length, stride length, base of support, swing time, and regularity index. This is in contrast to other members of the gp130 family of cytokines including IL-6 and LIF, which have demonstrated significant effects on motor recovery. Mice treated with an antibody against the IL-6 receptor were found to have significantly better functional recovery compared to control mice and reduced glial scar tissue [7]. Mice treated with LIF have demonstrated increases in proliferation of microglia/macrophages that corresponded with significant decreases in motor function compared to control mice [11]. This is in contrast to another study that found significantly better motor performance in mice that received intraperitoneal delivery of LIF relative to minocycline- or albumin/PBS control-injected mice after hemisection cord injury [30]. It is unclear as to exactly why IL-11 signaling does not appear to play as significant role in affecting functional recovery after SCI. One possibility, as stated beforehand, could be the later increase in expression of IL-11Rα after SCI, which limits IL-11 signaling in the spinal cord until later time points after injury. Both IL-6 and LIF gene expression peak in the more acute phases after injury with IL-6 and LIF peaking at twelve hours and six hours, respectively. While the gene expression of the receptors for IL-6 and LIF have not been documented and thus cannot directly be compared with that of IL-11, the peak gene expression of IL-11 at 24 hours post-SCI is still later than either IL-6 or LIF, suggesting that IL-6 and LIF, in combination with other pro-inflammatory cytokines, may play more dominant roles in the early setup of the molecular and cellular environment in the spinal cord after injury before IL-11 signaling can come into effect.

In support of the lack of significant differences in motor recovery between wild-type and knockout mice, we found no significant differences in axonal conduction and histopathology in the injured spinal cord. This supports the idea that IL-11 may not play as dominant a role in the early inflammatory signaling after injury as other pro-inflammatory cytokines and other cytokines in the gp130 family, especially with similar amounts of scarring between wild-type and knockout mice. However, this finding is in contrast to previous studies demonstrating a role for IL-11 in promoting oligodendrocyte viability and maturation *in vitro*[16,17]. Based on these studies, it was originally hypothesized the IL-11Rα knockout mice would demonstrate poorer axonal conduction and white matter sparing relative to wild-type mice. However, the absence of significant differences between injured wild-type and knockout mice suggests the presence of a protective factor. Mice treated with LIF have demonstrated decreases in oligodendrocyte apoptosis after SCI [10,12]. In this study, LIF may have provided the protective signaling to prevent further demyelination and loss of function after SCI. This suggests redundancy of function of the gp130 cytokines in SCI and perhaps points to an innate protective mechanism to prevent further damage should one cytokine pathway be nonfunctional.

There has been extensive debate about the use of knockout models in addressing injury to the central nervous system. This has been demonstrated most recently with the conflicting results surrounding the effect of Nogo, myelin-associated glycoprotein (MAG), and oligodendrocyte myelin glycoprotein (OMgp) in axonal regeneration after injury to the brain and spinal cord based on genetic knockout mice models [31]. It is possible that knocking out the IL-11Rα gene in the embryonic stage may have resulted in developmental compensation by other cytokines in the gp130 family that may have masked any potential detrimental effects ablating IL-11 signaling may have in SCI. Thus, it is important to note that although the results of this study did not demonstrate significant functional differences between knockout and wild-type mice, IL-11 may still play an important role in the pathophysiology of spinal cord injury that were not detected in this study.

To further address the role of IL-11 signaling in SCI, it is suggested that pharmacological methods be implemented. Studies addressing both pharmacological inhibition of IL-11 or IL-11Rα, using neutralizing antibodies, or overexpression/administration of IL-11 should be carried out to see if differences in functional recovery and histopathology may occur after SCI. In this case, the question of compensation present in the genetic model can be circumvented. Further studies characterizing the role of IL-11 signaling in oligodendrocyte viability, maturation, and myelin formation in *in vivo* models of SCI would also provide valuable insights into the role of this cytokine.

To date, this is the first study to examine the potential role of IL-11 signaling in the pathophysiology of SCI. Despite the lack of significant differences in functional recovery after SCI between wild-type and knockout mice, clear changes in expression of IL-11 and its α-receptor suggest that IL-11 signaling plays a role in SCI. The results of this study suggest that IL-11 does not play a major role in the pathophysiology of SCI but, instead, plays a role secondary to other cytokines including TNFα, IL-1β, IL-6, and LIF and that there is redundancy in the spinal cord cytokine network. The timing of expression is a key factor in determining the ultimate role of a cytokine after SCI, especially in relation to other cytokines and in determining which cytokines’ effects will predominate in the pathophysiology of the injury. Possible developmental compensation in the knockout model used for the study suggests that we must exercise caution in concluding that the null results of this study suggest that IL-11 does not play a role in SCI. Future studies can bypass this limitation through the use of pharmacologic inhibition of both IL-11 and its receptor.

This study demonstrates that despite sharing a common signal transducing receptor, members of the gp130 family of cytokines play different roles in SCI. Future therapies should focus on modulating the cytokines shown to have significant ramifications on functional recovery as these will likely result in the greatest yield. As we begin to understand the major players in the pathophysiology of SCI, we can move forward to design more specific therapies to improve functional recovery.

## Conclusions

Our study suggests that IL-11 signaling may not play a major role in the pathophysiology of SCI. This study is the first study to address the role of IL-11 in SCI and hopefully will provide a basis for future research in understanding the role of the gp130 family of cytokines in inflammation after injury. Ultimately, this understanding will hopefully lead to better and more specific therapies aimed at modulating cytokine signaling and decreasing damaging inflammatory processes after SCI.

## Competing interests

The authors declare that they have no competing interests.

## Authors’ contributions

NC performed all of the surgeries, contributed to the design of the study, drafted the manuscript, and performed all experimental procedures, data acquisition, and statistical analysis except for electrophysiological recordings. DHN contributed to the design of the study and participated in the collection of behavioral data, qRT-PCR data, histology data, and myeloperoxidase data. KS performed the electrophysiological recordings and associated data analysis. DRB contributed to the design and coordination of the study. MGF conceived the study and directly supervised all aspects of the design, coordination and analysis of the study. All authors read and approved the final manuscript.
